# In this Issue: Volume 113, Issue 10, October 2022

**DOI:** 10.1111/cas.14962

**Published:** 2022-10-04

## Mechanisms of resistance to immune checkpoint inhibitors


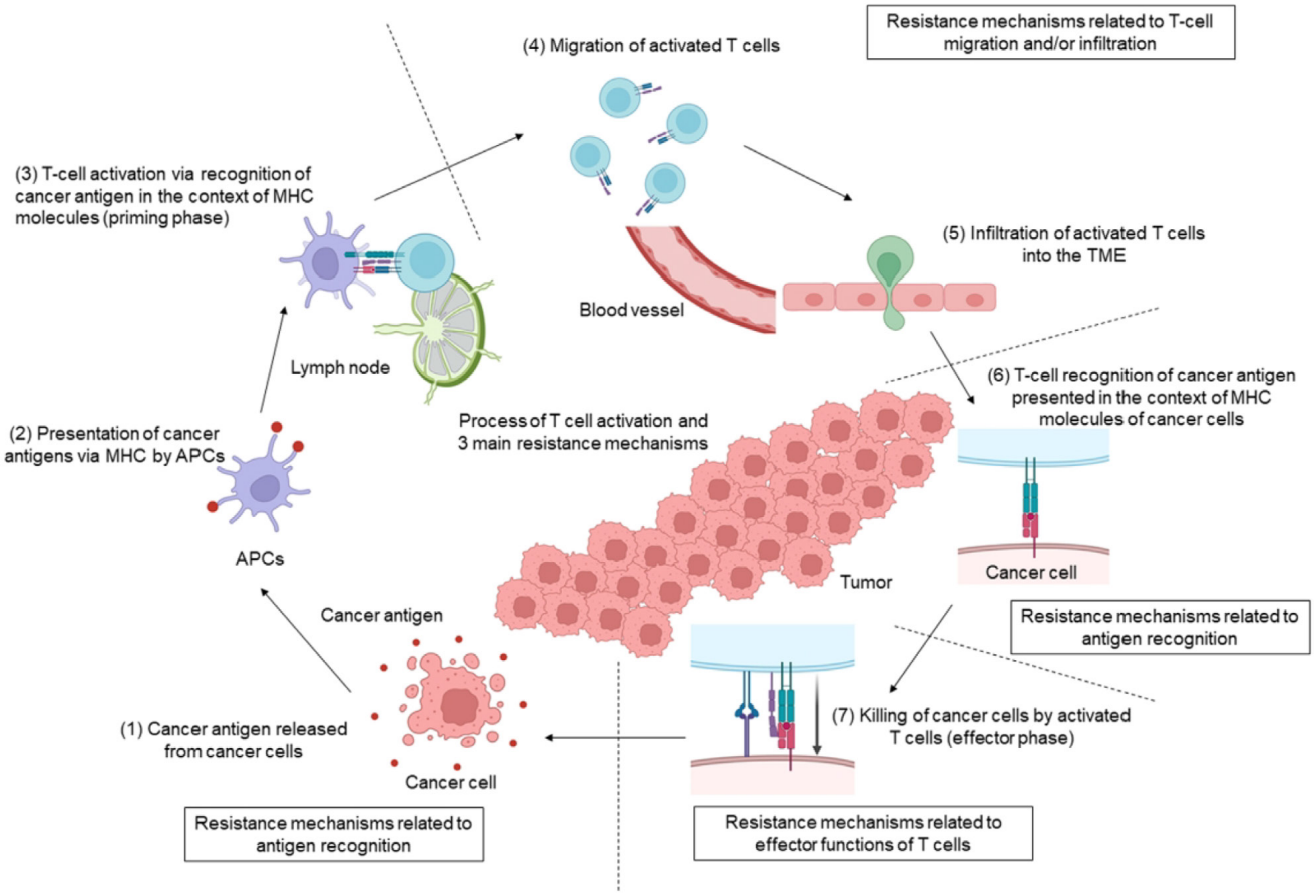


The introduction of immune checkpoint inhibitors (ICIs) has reshaped the course of cancer treatment. Immune checkpoint inhibitors are drugs used in the immunotherapy of cancer patients. They inhibit signals that suppress the activity of immunogenic T cells, thereby promoting T cell‐mediated antitumor effects. This proves to be a huge asset to the clinical treatment of cancer, but the fact remains that many patients show or acquire resistance to ICIs during the course of treatment. Thus, to improve clinical outcomes, it is important to explore the resistance mechanisms that render this novel therapy ineffective.

To this end, Nagasaki et al. detail the resistance mechanisms that work against the T cell‐activating ICIs and broadly classify them into three major categories.

In the first category, resistance is attributed to insufficient antigen (a foreign body that elicits an immune response) recognition by the body’s immunogenic T cells, leading to impaired activation of these cells. This mechanism is grounded in the concept that the initial steps of T cell activation involve their recognition of cancer antigens, a process aided by antigen‐presenting cells. After their recognition of the cancer antigen, the now activated T cells migrate and infiltrate the tumor microenvironment (TME) and recognize specific cancer antigens. Any roadblock in this process (or the signaling pathways involved in it) gives rise to a second category of resistance, which is attributed to the dysfunctional migration and infiltration of T cells into the TME. Finally, the cytotoxic effects of T cells are used to destroy cancer cells. Any issues in this process give rise to a third category of ICI resistance, caused by the reduced functioning of T cells. Certain mutations (like those of the gene responsible for production of Janus tyrosine kinase, a regulatory protein) cause abnormal γ‐interferon (IFN‐γ, a cytokine secreted by activated T cells to confer antitumor immunity) signaling, resulting in ICI resistance. This abnormal signaling was found to hinder T cell antigen recognition through the decreased expression of MHC‐I molecules on cancer cells and their migration and/or infiltration into the TME through decreased expression of chemokines and also to attenuate inhibitory effects of IFN‐γ on cellular proliferation.

Resistance to ICIs stems from a complex interplay of factors and mechanisms. However, it can be controlled using different drugs. Various combinations of mAbs are being explored to block T cell‐suppressing interactions between the immune checkpoint molecules, so that T cells remain activated. Finally, immune cell therapy successfully treated ICI‐resistant cancers, such as malignant lymphoma and leukemia, opening doors for its future application in treating other tumors.

https://onlinelibrary.wiley.com/doi/10.1111/cas.15497

## Tumor cell–released LC3‐positive EVS promote lung metastasis of breast cancer through enhancing premetastatic niche formation


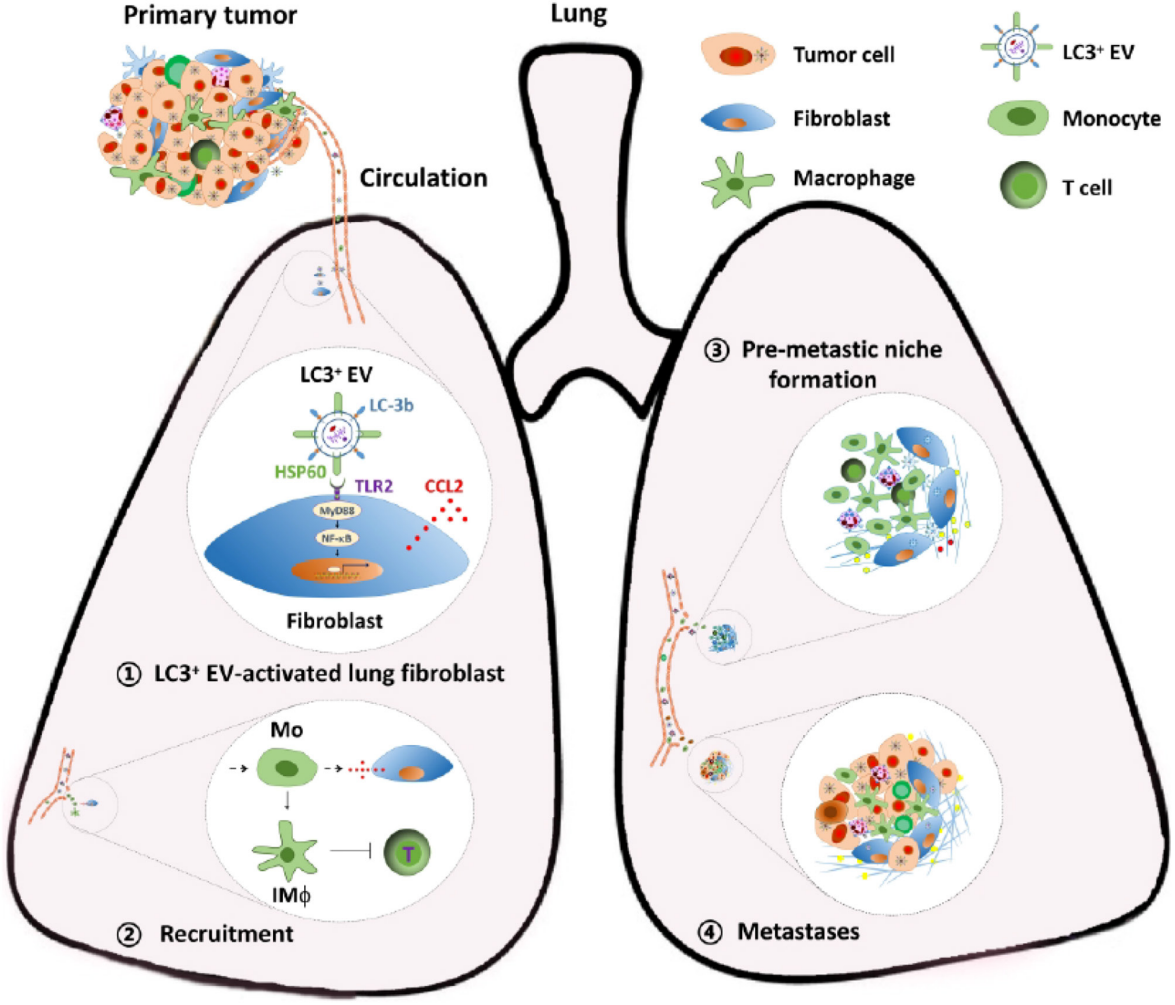


Breast cancer (BC) is the most commonly diagnosed type of cancer among women across the world. Most BC‐associated deaths are a result of distant organ metastasis, that is, the migration and invasion of tumor cells from the breast to other organs. The lungs, in particular, are the most prone to distant metastasis due to BC. Unfortunately, lung metastasis is highly difficult to treat, resulting in significant mortalities.

Distant organ metastasis relies on the accumulation of circulating tumor cells (CTCs) in the distant organ, made possible by the premetastatic niche (PMN), an environment that supports the colonization and survival of CTCs. Hence, reducing PMN formation can help minimize the growth of malignant BC tumors.

Premetastatic niche formation depends on several tumor‐secreted factors, including LC3^+^ extracellular vesicles (EVs), lipid bilayer‐delimited particles that travel to the site of metastasis and regulate PMN formation. Specifically, LC3^+^ EVs regulate the actions of immune cells that suppress their ability to fight cancer cells in the tumor environment. However, their exact role in PMN formation is unclear.

This study aimed to clarify the role of BC‐released LC3^+^ EVs in the development of lung metastasis based on experiments on mice models. Findings indicated that, first, lung fibroblasts of the mice were activated by LC3^+^ EVs to increase the production of C–C motif chemokine ligand 2 (CCL2), a chemokine ligand that attracts monocytes and other important immune cells to the site of an injury.

This activation was brought about by heat shock protein (HSP) 60—a protein present on the surface of LC3^+^ EVs—through a signaling cascade in the lung fibroblasts. Next, CCL2 attracted monocytes to the lung fibroblasts, leading to the suppression of T cells with anticancer activity, thus marking the start of PMN formation. As expected, a reduction in LC3^+^ EV and HSP60 levels or the inhibition of CCL2 led to the suppression of PMN formation and lung metastasis.

The study also found that there was an increase in LC3^+^ EV levels and HSP60 levels on LC3^+^ EVs in the plasma of patients with BC, indicating its strong association with the progression of lung metastasis. Hence, these two factors can serve as important biomarkers of lung metastasis resulting from BC.

These findings can guide the development of new therapies that target these biomarkers and provide effective treatment to patients with metastatic BC in the future.

https://onlinelibrary.wiley.com/doi/10.1111/cas.15507

## N4BP3 promotes angiogenesis in hepatocellular carcinoma by binding with KAT2B


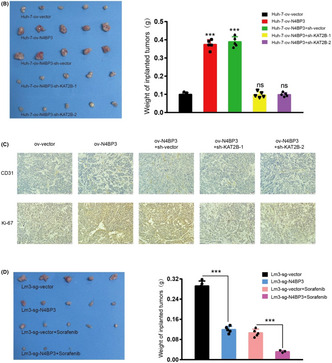


Hepatocellular carcinoma (HCC), the most common type of primary liver cancer, is one of the most common malignant tumors of the digestive system. It is the second leading cause of cancer‐related deaths globally. Hepatocellular carcinoma has a high incidence rate and often recurs even after aggressive local therapies, emphasizing the need to discover and develop new methods to treat this disease.

Tumors in HCC are frequently associated with an abundance of new blood vessels, due to which radical surgery is not an option for most patients. Recently developed antivascular molecular‐targeted therapy is a potential treatment strategy for patients who have lost the chance for radical surgery.

Sorafenib, an orally administered new class of targeted drug, has become the standard treatment for advanced‐stage HCC in recent years. However, sorafenib is associated with various side‐effects, including diarrhea, increased blood pressure, and skin lesions. Moreover, the high cost and limited improvement in patients’ survival limit its use. There have been efforts aimed at improving the sensitivity of targeted drugs.

In this study, Han et al. found NEDD4 binding protein 3 (N4BP3) as a novel pro‐angiogenic factor in HCC and undertook multilayer experiments in cells, animals, and tissues to investigate the mechanisms involved in forming new blood vessels (angiogenesis) with HCC development.

Using the *N4bp3* KO mouse model, they found that N4BP3 plays an important role in developing and progressing HCC and promoting angiogenesis in HCC. They then analyzed the specific mechanisms through which N4BP3 promotes angiogenesis in HCC.

Cell biology experiments showed that when N4BP3 is suppressed in HCC cells, the formation of the blood vessels is reduced, while overexpression has the opposite effect. Further cell and molecular biology experiments have revealed that N4BP3 interacts with KAT2B (lysine acetyltransferase 2B), increasing signal transducer and activator of transcription 3 (STAT3) expression by regulating the distribution of acetyl‐histone H3 (Lys27) (H3K27ac) in its promoter region. This, in addition, regulates the activity of the STAT3 signaling pathway, which promotes the proliferation of microvessels in HCC and accelerates the malignant process of the tumor.

In vivo experiments in nude mice have confirmed their findings and suggested that N4BP3 could be a potential target for the treatment of HCC in combination with sorafenib.

https://onlinelibrary.wiley.com/doi/10.1111/cas.15498

